# Does candidate race influence simulated patient ratings in standardised assessments of clinical practice? A single-blinded randomised study in UK medical schools

**DOI:** 10.1136/bmjopen-2023-080543

**Published:** 2025-01-15

**Authors:** Celia Brown, Ann Sebastian, Sarah Khavandi, Kerry Badger, Rachel Westacott, Malcolm Reed, Mark Gurnell, Amir H Sam

**Affiliations:** 1 Warwick Medical School, The University of Warwick, Coventry, UK; 2 Imperial College London, London, UK; 3 University of Birmingham, Birmingham, UK; 4 Brighton and Sussex Medical School, Brighton, UK; 5 Wellcome Trust-MRC Institute of Metabolic Science, University of Cambridge, Cambridge, UK

**Keywords:** Stereotyping, MEDICAL EDUCATION & TRAINING, Patient-Centered Care, Patient Satisfaction

## Abstract

**Abstract:**

**Objectives:**

Standardisation of medical examinations involves minimising assessor stereotyping and bias for a fair process. This study aimed to determine whether being a non-white candidate affected scoring by simulated patients, compared with a white candidate, at three different performance grades in the same history-taking station.

**Design:**

Single-blinded, video-based, randomised study.

**Participants:**

163 simulated patients watched a randomly allocated set of six videos. Each set consisted of three white and three non-white (South Asian, black and Chinese) candidates performing at either fail, borderline or pass grades. Therefore, each simulated patient assessor observed one white and one non-white candidate at each grade and scored communication and professionalism domains.

**Main outcome measure:**

The median and interquartile range of the difference between total scores for the white and non-white candidates were compared at all three performance grades.

**Results:**

The black fail candidate scored statistically significantly lower than their white fail counterpart. The black borderline and Chinese borderline candidates scored significantly higher than their white counterparts. No other differences were statistically significant at p<0.0057.

**Conclusions:**

Being a black candidate at the fail level of performance was associated with a lower score than being a white candidate at the fail level of performance, thereby indicating a negative stereotype against black students. However, being black or Chinese at a borderline grade was associated with higher scores than being white candidate at the same grade potentially due to self-awareness of potential bias when there is uncertainty regarding the performance.

**STRENGTHS AND LIMITATIONS OF THIS STUDY**

- The study investigated simulated patient assessor bias across different races and levels of performance in medical students.
- The study is designed to mimic ‘real-life’ clinical examinations as much as possible.
- There was only one candidate per race, so the findings may not be generalisable to all within the race.
- While all the simulated candidates reflected similar performances within each grade, there may be a degree of variation in the performance.

## Introduction

 Summative assessments within undergraduate medical education, particularly in the final year, are designed to enable robust demonstration of a minimum level of competence, with a view to future safe practice by newly qualified doctors.^1^ To maximise the validity of these assessments, it is important to include assessors with different perspectives,^2^ whereas to maintain standardisation and fairness, it is important to minimise errors, stereotyping and other biases by assessors.

In medical school assessments, simulated patients (SPs) can replicate real-life scenarios in a controlled environment.^3^ SPs are used widely in clinical skills assessments and can provide useful and reliable insight into a candidate’s performance, particularly in areas such as communication and professional skills.^4^ Differing viewpoints between clinical assessors and SP assessors can serve as an important trigger for the clinician to reappraise the consultation from a lay person’s perspective.^5^

Previous studies have demonstrated both positive and negative stereotype biases by SPs. For example, certain physical attributes, including extravagant hair colours and tattoos have been associated with positive/no bias in some studies,^6^ but negative bias in others.^7 8^ Similarly, the presence of a regional (UK—Liverpudlian) accent was linked with bias in one experiment^9^; however, no bias for this accent was seen in another study.^6^

The potential effect of student race on examiners’ ratings of student performance has also been a focus of interest. Again, divergent perspectives have been reported, for example, some papers concluded the absence of influence on scores awarded by clinical assessors^10^ despite stereotype activation, whereas others have advanced the concept of ‘differential attainment’ in which ethnic minority groups are awarded lower scores than their white counterparts because of negative bias.^11^ It is clearly important to understand potential inequalities, as ethnic minority groups may have 2.5 times higher odds of failing examinations at medical school, and this leads to worse outcomes on graduation.^11 12^

To date, there is mixed evidence with respect to the effect of candidate race on scores awarded by SP assessors in examinations. Some studies have demonstrated no effect on overall scores and interpersonal skills scores,^13^,^15^ and the candidate having the same race as the SP did not confer an advantage.^16^,^18^ However, other studies have demonstrated positive bias by SPs towards white students compared with other ethnicities such as Asian American students when assessing empathy^19^ and lower overall scores for black/African American students.^20^ The evidence base is therefore mixed, and results may be affected by context, including the grade at which the student is performing. Therefore, we aimed to investigate the potential influence of candidate race on the scores awarded by SPs to medical students performing at various grades. To do this, volunteer SPs were invited to assess the performances of simulated candidates using an online platform, with candidates of varying races exhibiting different, standardised performance grades.

## Methods

### Study design

The study used a single-blinded, video-based, experimental, randomised design and was delivered digitally.

### Setting and participants

SPs were invited to participate via email, distributed by the Professional Role Players company who hold a database of SPs involved with teaching and assessment in UK medical schools. Accordingly, SPs were recruited by an independent organisation from across the UK, helping to reduce participation bias. Recruitment and data collection took place between October 2021 and February 2022. To avoid bias, SPs were informed that the study aimed to investigate inter-rater reliability among SPs. They were not informed that it also aimed to evaluate the effect of candidate race on the scores awarded by SPs. Participants were able to undertake the study in their own time at a location of their choice.

### Variables and data measurement

Twelve 10 min videos were created of simulated candidates taking a focused clinical history from an SP with symptoms of cholecystitis, presenting their findings, reviewing investigations and proposing a management plan.

The videos consisted of six candidates from four different ethnicities. Three candidates were from the white group, and three were from the non-white group consisting of Asian (also referred to as South Asian, indicating people of Indian, Pakistani or Bangladeshi descent), black and Chinese ethnicities. Each of the three white simulated candidates performed the station at one of the three overall performance grades: fail, borderline or pass. The remaining nine videos were of each of the three non-white simulated candidates performing at each of the three grades.

Three sets of six videos were developed. Each set consisted of the three white simulated candidates performing at one of the fail, borderline or pass performance grades. These videos remained constant across all the sets. The remaining three videos consisted of each non-white simulated candidate performing at one of the fail, borderline or pass performance grades; thus, each white candidate was paired with one non-white candidate performing at the same grade. The pairings varied between sets so that each race was represented at each performance grade (table 1). Each set had four different randomised orders of the six videos to minimise any bias associated with ordering effects. The experienced SP was the same in all videos.

**Table 1:** Video sets

Video set 1	White pass	White borderline	White fail	Chinese pass	Black borderline	Asian fail
Video set 2	White pass	White borderline	White fail	Chinese borderline	Black fail	Asian pass
Video set 3	White pass	White borderline	White fail	Chinese fail	Black pass	Asian borderline

The simulated candidates included clinical teaching fellows of the Imperial College School of Medicine and clinical staff of the Imperial College Healthcare Trust. All candidates were men and of a similar age. Although all candidates spoke English fluently, the Chinese candidate had a mild Chinese accent, whereas the black, Asian and white candidates spoke with received pronunciation.

**Figure 1:**
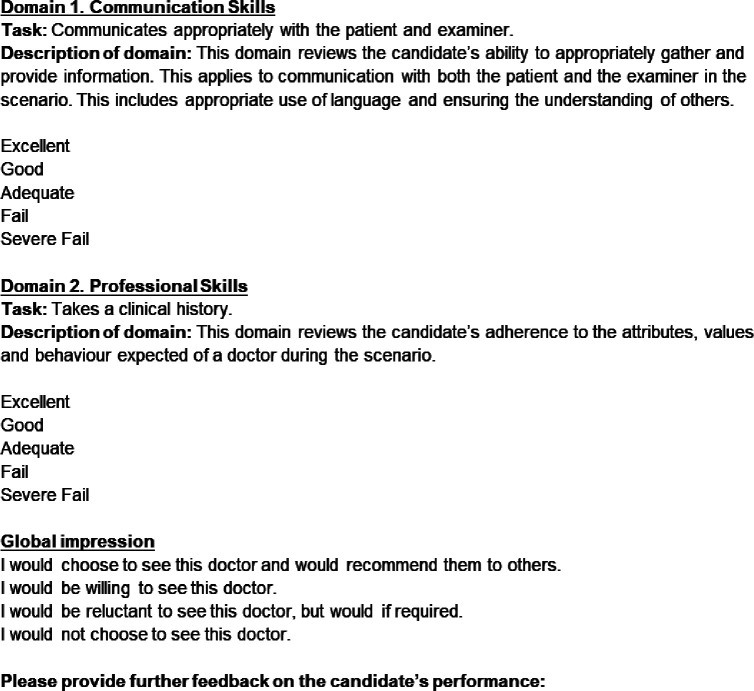
Sample marksheet.

Each SP was randomly allocated one of the video sets to watch on the online platform. They viewed one video at a time and marked that candidate simultaneously using an online marksheet (figure 1). Each candidate was assessed at the level expected of a newly qualified Foundation Year 1 doctor. They were assessed on performance in two domains (communication and professional skills). Within each domain, the SP had to choose between severe fail, fail, adequate, good and excellent, and these were transposed into numerical values between 0 and 4, giving a total possible score range of 0–8. SPs also chose an overall global impression for each candidate from one of the following: ‘I would not choose to see this doctor’, ‘I would be reluctant to see this doctor, but would if required’, ‘I would be willing to see this doctor’ or ‘I would choose to see this doctor and would recommend them to others’. Each global impression option was assigned a number from 1 for ‘I would choose to see this doctor and would recommend them to others’ to 4 for ‘I would not choose to see this doctor’. They were also provided with a free text box to allow further written feedback on the candidate’s performance if they felt this to be appropriate. To mimic the clinical examination setting, participants were unable to rewind, replay or pause videos. However, they were able to return to marksheets of previous candidates. Following completion of the six videos, the participant completed a brief questionnaire on their previous experience as an SP in medical school examinations and clinical teaching, their sex, ethnic background and geographic region of their work in the UK.

SPs were provided with a participant information sheet to read and were asked to consent to the recording of their results anonymously by digitally signing via a tick-box. Participants were able to withdraw from the process via an opt-out button in which case their data were not recorded. However, it was not possible to withdraw retrospectively (ie, after submitting results and demographic information) owing to the lack of identifiable information.

### Study size

The study aimed to compare white candidates with each of the other three races at each performance level (but not to compare across the three non-white races), and the required sample size calculation for this analysis was performed on Stata V.17. The study had a medium effect size of 0.5 SD, power of 80% and critical p-value of 0.0057 due to the multiple pairwise comparisons using the Sidak correction. The minimum sample for the nine paired comparisons required 53 participants for each of the sets of three comparisons and, therefore, 159 altogether.

### Statistical methods

Data management and analysis were conducted on Stata V.17. For each participant, as paired data, the difference was calculated between the total score awarded to each of the non-white (Asian, black and Chinese) and each of the white candidates at fail, borderline and pass performance grades, respectively. The median and IQR of the difference in the total score between the white and non-white candidate at each of the fail, borderline and pass performance grades were calculated. Individual Wilcoxon matched-pairs signed-rank tests were conducted to investigate the significance of these differences. The Sidak correction for multiple pairwise comparisons was applied and a p value of <0.0057 was required for significance (table 2). Global impression scores were also compared between each non-white and white candidate at each performance grade. The significance of these differences was calculated using Wilcoxon matched-pairs signed-ranks tests.

**Table 2:** Number of simulated patients (SPs), median, and 25th and 75th percentiles of the difference between each of the non-white (Asian, black and Chinese) and white candidate pairs at each performance grade

Total score of non-white (NW) candidate − Total score of W candidate	SPs (n)	Median difference	25th percentile difference	75th percentile difference	Total score of NW versusTotal score of W (z score)	Total score of NW versusTotal score of W (p-value)	Conclusion
Fail							
Asian (A) − White (W)	51	0	−2	1	1.143	0.2531	No difference
Black (B) − W	52	−1	−2	0	4.878	**<0.001**	**Black fail scores lower**
Chinese (C) − W	60	0	−1	1	0.624	0.5325	No difference
Borderline							
A − W	60	1	−1	2	−2.550	0.0108	No difference
B − W	51	2	0	2	−5.624	**<0.001**	**Black borderline scores higher**
C − W	52	1	0	2	−4.173	**<0.001**	**Chinese borderline scores higher**
Pass							
A − W	52	0	−1	0	2.693	0.0071	No difference
B − W	60	0	0	0	0.818	0.4132	No difference
C − W	51	0	−1	0	0.724	0.4692	No difference

Bold font: p-values and corresponding conclusions in bold font show those results statistically significant at p<0.0057.

### Patient and public involvement

Patients and/or the public were not involved in the design, or conduct, or reporting, or dissemination plans of this research.

## Results

**Figure 2:**
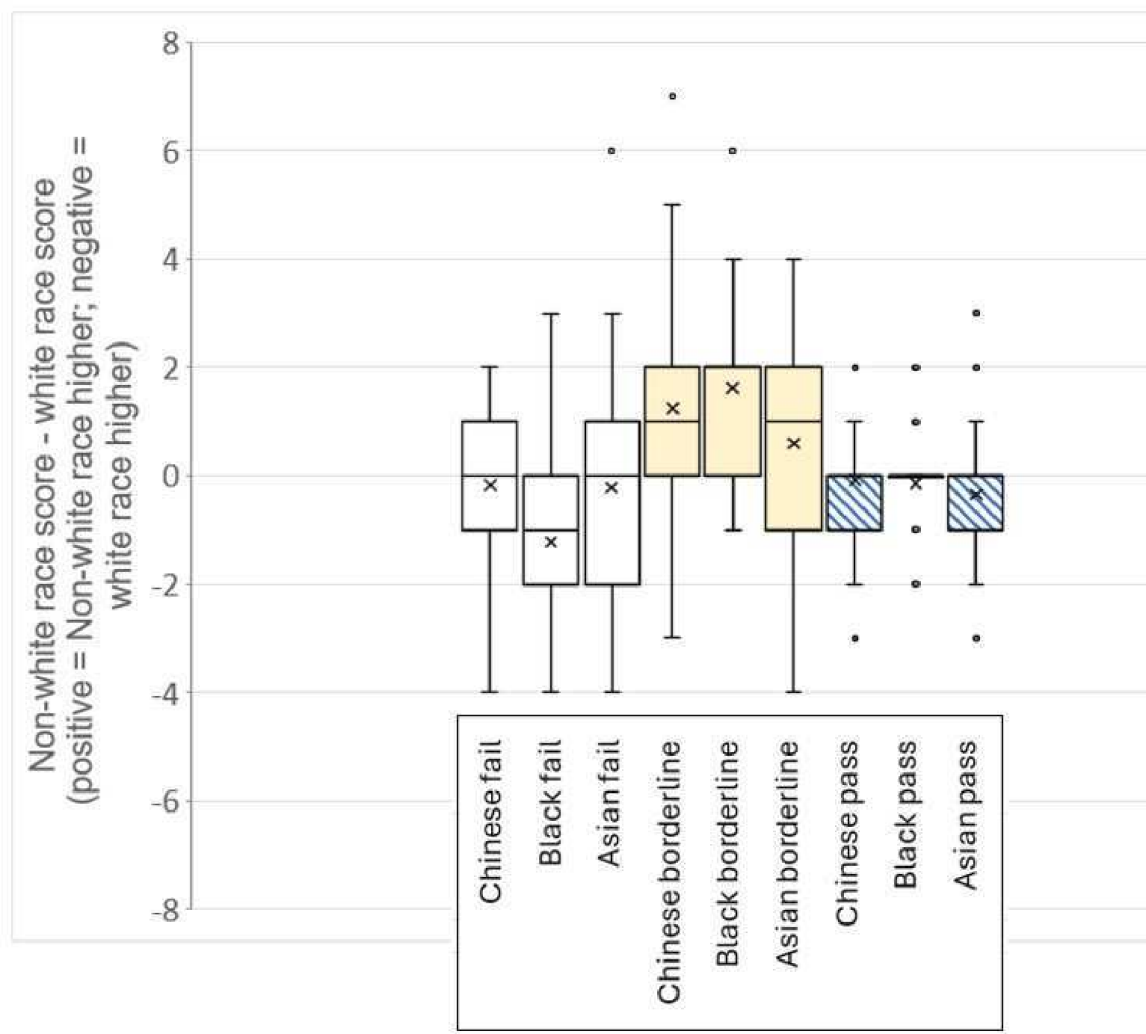
Absolute difference between the non-white race candidate at each performance level and their paired white candidate at the same performance level. The ‘x’ in each box represents the mean difference.

163 SPs participated in the study, all of whom were included in the analysis. Previous experience, ethnic background and region of work in the UK varied across participants (online supplemental figure 1). Full results—comparing each non-white candidate to their white counterpart—are shown in table 2. The black fail candidate scored significantly lower than their white fail counterpart. In contrast, the black and Chinese borderline candidates scored significantly higher than their white counterparts (figure 2). For the remaining candidates, no significant difference in scores was found between the paired non-white and white candidates at each grade (figure 2).

The comparisons of ‘global impression’ scores concurred with the results seen for the difference in total scores between the non-white and white candidates. The black fail candidate had significantly worse global impression scores overall compared with the white fail candidate (z score −3.008, p=0.0026). The black borderline and Chinese borderline candidates had better global impression scores than the white borderline candidate (z score 4.935, p<0.001 and z score 3.712, p<0.001, respectively).

## Discussion

We investigated whether the race of medical students performing at various grades influenced the scores awarded by SPs in clinical examination stations testing communication and professional skills. This study found that the SPs gave significantly lower scores to the black fail candidate than the white fail candidate. Although both were scripted to perform at the same grade, the median difference in the scores awarded to these candidates was large (1 out of 8 marks). In contrast, the black borderline and Chinese borderline candidates were given higher scores than the white borderline candidate. Again, there was a large median difference between both white borderline and each of black borderline and Chinese borderline candidates (1 out of 8 marks for both). No significant differences were found between the scores of each of Asian fail and Chinese fail and the white fail candidate although the trend was towards lower awarded scores for the non-white candidates than that for white candidates.

Across the literature, studies have shown that non-white candidates achieve lower scores compared with their white counterparts. This has been the case even when previous examination achievement, type of examination or study habits have been controlled for.^21 22^ The effect extends beyond medical school, such as in communication skills stations in postgraduate exams.^23^ Several reasons have been suggested for this difference. For example, one theory is that it is not necessarily bias that results in such differences in scores; rather, such differences are attributable to cultural differences and language barriers.^24^ This study minimised any bias based on these factors by using scripts for both the student and the SP thereby standardising performance across candidates.

In this study, among the non-white groups, only the black fail candidate scored significantly lower than the white fail candidate performing at the same grade, implying a bias against black candidates. Concerningly, this has been seen in previous studies where black or African American students have received lower scores for empathy from SPs when compared with their white counterparts.^20^ Another study found that SPs scored black students and Asian students lower than their white counterparts, particularly in the communication section.^25^ Although studies have shown that Asian students receive lower scores than their white counterparts,^24 26^ this was not reflected in the scores of either the Asian or Chinese candidates at any of the performance grades in this study. However, it is difficult to determine if there is bias given that only a single candidate was used to represent each of South East Asia and China. It is challenging to compare between studies given that the use of the description ‘Asian’ differs across the literature - this has not been explored in this study. Although the black fail and white fail candidates both failed the station included in this study, receiving a significantly lower failing score would affect the overall mark and therefore could alter pass/fail decisions, particularly if this finding is reflected across all clinical assessments in medical schools. It is essential to increase awareness of such bias and provide appropriate training for SPs and examiners.

In contrast, the higher scores given to black borderline and Chinese borderline candidates as compared with the white borderline candidate is a novel finding. For borderline candidates, where there is uncertainty about the quality of the performance, non-white candidates may be given the benefit of the doubt when compared with their white counterparts to compensate for any potential bias. This may be due to increased self-awareness following Equality, Diversity, and Inclusion training provided to SPs particularly given the recent drive to tackle racism in higher education.^27^ In contrast, such self-awareness might not surface when the candidate has performed so poorly as to merit the award of a clear fail. However, whether these findings would be consistent in all stations is unclear, as the definition of the ‘borderline’ candidate is case specific with a high degree of disagreement on fail/borderline/pass among examiners across all stations.^28^ Therefore, the effect of race on the borderline cohort would require further research expanding beyond one station and with various candidates demonstrating various levels of what is considered ‘borderline’. Interestingly, our findings align with the results of another study investigating the influence of candidates’ race on examiners’ ratings in standardised assessments of clinical practice.^29^

### Limitations

The study used videos of simulated candidates, so it is unclear whether SPs would demonstrate a similar level of bias during an in-person clinical examination, which is the usual method of assessment. In addition, one candidate was employed for each non-white category. Future studies should include more non-white candidates from a wider range of countries. The Chinese candidate had a mild Chinese accent, whereas the black, Asian and white candidates spoke with received pronunciation. Therefore, the separate effects of visual and aural racial cues could not be determined. Furthermore, we controlled for sex but were unable to control for all variables such as appearance and behaviour.

Although the simulated candidates reflected similar performances within each grade (fail/borderline/pass), as the performances were scripted, there could have been a degree of variation in the performance. Therefore, we sought to address this important possible confounder by reviewing the individual videos of the candidates performing at the borderline and fail grades. Specifically, we looked for subtle differences in performance between the black fail and white fail candidates and between the white borderline, black borderline and Chinese borderline candidates. However, we were unable to identify any differences in performance that would be expected to translate into differences in SP scoring for these candidates.

Future studies could explore whether the race of SPs influences the observed differences. Further studies could also assess the effect of both candidate race and sex on scores.

## Conclusion

In this study, SP assessors demonstrated a negative stereotype bias against black fail candidates and, in contrast, a positive stereotype bias towards both the black and Chinese borderline candidates, when compared with their white counterparts.

## supplementary material

10.1136/bmjopen-2023-080543 online supplemental file 1

## Data Availability

The raw scores awarded by each participant for each video are available on request from the corresponding author. Data on participant demographics are not available at individual level to protect the identity of participants.
