# Supplementary material for: Does candidate race influence simulated patient ratings in standardised assessments of clinical practice? A single-blinded randomised study in UK medical schools
Source: BMJ Open. 2025 Jan 15;15(1):e080543. doi: 10.1136/bmjopen-2023-080543 (PMC11751911; doi:10.1136/bmjopen-2023-080543)
Supplement: online supplemental file 1 [file bmjopen-15-1-s001.docx]

**Supplemental material**


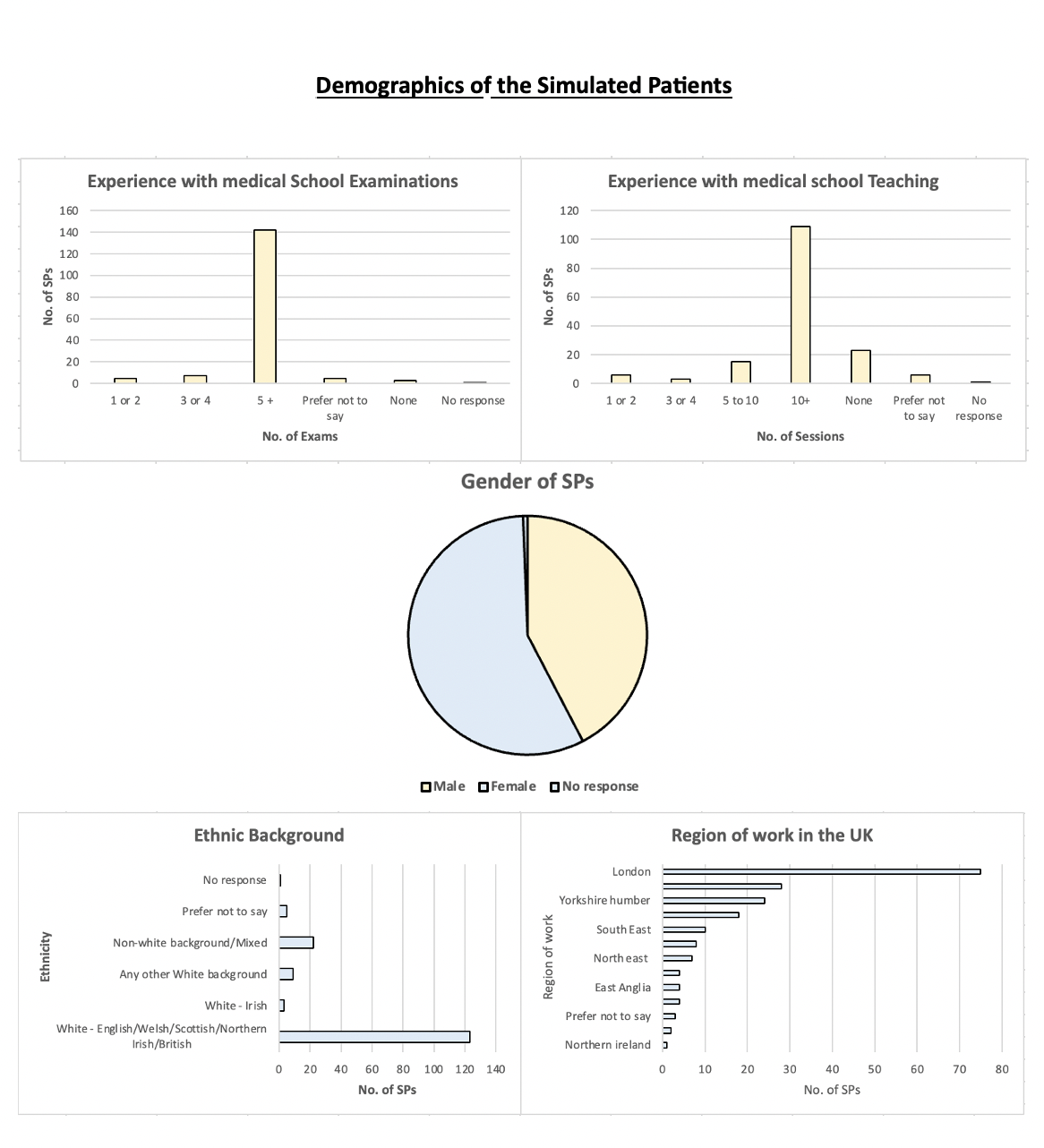


**Supplemental Figure 1.** Simulated Patient (SP) demographic informaion including experience with medical school examinations and teaching, gender, ethnic background and region of work in the UK.
