# Supplementary material for: Does candidate race influence simulated patient ratings in standardised assessments of clinical practice? A single-blinded randomised study in UK medical schools
Source: BMJ Open. 2025 Jan 15;15(1):e080543. doi: 10.1136/bmjopen-2023-080543 (PMC11751911; doi:10.1136/bmjopen-2023-080543)
Supplement: Supplementary file 2 [file bmjopen-2023-080543.reviewer_comments.pdf]

PEER REVIEW HISTORY

BMJ Open publishes all reviews undertaken for accepted manuscripts. Reviewers are asked to complete a checklist review form and are provided with free text boxes to elaborate on their assessment. These free text comments are reproduced below.

ARTICLE DETAILS

Title (Provisional)

Does candidate race influence simulated patient ratings in standardised assessments of clinical practice? A single-blinded randomised study in UK medical schools.

Authors

Brown, Celia; Sebastian, Ann; Khavandi, Sarah; Badger , Kerry; Westacott, Rachel; Reed, Malcolm; Gurnell, Mark; Sam, Amir H.

VERSION 1 - REVIEW

**Reviewer**	**1**
**Name**	**Haile, Sarah**
**Affiliation**	**University of Zurich, Epidemiology, Biostatistics and Prevention Institute**
**Date**	**07-Dec-2023**
**COI**	**None declared**

My main comments relate to the choice of candidates. It's unclear why there are 3 white candidates but only 1 each of the non-white candidates. Could there not have been more non-white candidates? I also question the choice of Asian, Black and Chinese. Is Chinese not Asian? Of course Asian could mean many things, and is a very diverse group. Why not latino or middle eastern? It was also noted that the Asian and Black candidates did not have an accent when speaking but that the Chinese candidate did. This seems like a poor choice of candidates, and makes it difficult to state that race was the sole explanation for the difference, when attitudes about language fluency and intelligibility would also play a role here.

Was there sufficient sample size to distinguish between the 3 non-white groups?

Regarding Table 1: I understand the rationale behind using median and IQR in this case. But I wonder if it glosses over small differences. Mean and SD might be more informative here. Also, a graphical display, e.g. a stacked barplot, might be a nice addition to understand the data.

I found the category names hard to follow. Perhaps B1, B2, and B3 or something like that? or change "BD" for borderline to something else.

"p< 0.00057" should probably contain an "="?

DEI/EDI usually contains the word equity not ethnicity doesn't?

Reviewer **2**

Name **Nishimura, Takeshi**

Affiliation **Hyogo Emergency Medical Center, Emergency and Critical Care Medicine**

Date **15-Jan-2024**

COI **none**

Authors described paper entitled "The influence of candidates' race on simulated patient ratings in standardised assessments of clinical practice: a single-blinded randomised study." This paper addresses the potential bias for race and is well written, however, some concerns are still remained.

Title, typo "standardized", "randomized"

Introduction is too redundant. It is unclear what is known and what is unknown, and what is the aim of this study. There is no clear statement why author performed this study.

Asia and Chinese look similar (China is located in Asia), what is the reason authors compared A and C separately.

Statistical method should be discussed, this reviewer is unsure Wilcoxon matched pairs signed rank test was appropriate in this study.

Also, there might be a problem in comparison, answer list in mark sheet should not be converted to discrete variable directly (0-8).

There is no reason why authors compared white and non-white separately (fail, borderline, and pass).

A various bias of simulated patients was not under consideration for comparison. For instance, when SPs were white, they might feel sympathy for white candidate. Furthermore, potential bias except for race such as behavior, appearance, looks, etc should be discussed and omitted as much as possible.

Supplement is duplicated. In addition, the legend in supplement file should be attached.

In discussion "The aim of this study was … communication and professional skills …the candidate". However, as authors stated, it is well known that race affect scores evaluated by assessors. Further information in detail about the aim of this study is necessary.

Some of the sentences were repeated, should be avoided.

“Our study found that the SPs gave significantly lower scores to the BF candidate as compared to the WF candidate” “the BBD and CBD candidates were given higher scores than the WBD candidate”, there is no explanation why these results were obtained in this study. Theoretical explanation is needed in discussion. Authors are just guessing without any supported evidence/data.

Overall, text and statistical method is complicated and involved sentence, more simple expression is preferable.

**Reviewer**	**3**
**Name**	**Poenaru, Dan**
**Affiliation**	**MyungSung Christian Medical Center, Pediatric Surgery**
**Date**	**05-Apr-2024**
**COI**	**none**

This is a randomised single-blinded study investigating the effect of race on clinical assessments by standardised patients. The authors are to be commended for a carefully conducted randomised study which employs solid methodology and attempts as much as possible to remove analytic biases and reach the appropriate conclusions.

Despite the well-conducted study, the results are limited in extent and also somewhat surprising. The fact that black students tend to be rated lower than white students by standardised patients is well known and indeed totally expected. However both the lack of similar findings for other races, as well as the very unusual finding of apparent reverse discrimination in the case of borderline students is difficult to explain. I am personally not convinced that the authors’ explanation of this last finding is tenable — ie that racial bias is moderated by the standardised patients in the specific case of borderline students — nor can any solid scientific conclusions be unfortunately drawn from this.

Ultimately, the limited findings of the study may point back to study limitations which were at least in part noted by the authors themselves. Despite the large number of videos included — as required by the sample size calculation for the randomization — the presence of only one person from each race definitely opens the results to validity questions. It is also surprising that all students were male, and this unusual choice is not explained in the methodology.

Another question which remains unanswered is the role of standardised patients’ demographics on their ratings. While these characteristics are described in the supplementary information, and there is, as expected, a certain unbalanced distribution of

races with the white one being predominant, no conclusions can be drawn on this important factor in the absence of any inferential or even descriptive analyses.

The ultimate question regarding this study is its significance and impact. If the unusual finding regarding borderline patients (which has never been previously reported in other studies) cannot be directly trusted in a programmatic fashion, and the finding that black students are generally scored lower is well-documented throughout the literature, what practical conclusions and implications are there from the study? As much as the results are interesting and partly support the existing literature, unfortunately I do not see what they actually add to the literature. I suspect that for the study to be practical, its size would need to be increased in terms of students actually being assessed, it would need to include more than one gender, correlate the findings with the demographics of the standardised patients themselves, and include a qualitative exploration of the reason why ratings were those observed.

Another area that requires exploration in light of this study is the type of assessments used by standardised patients. In the current study all of these assessments are purely subjective, almost “inviting” almost any inherent implicit biases to come out. Educational literature to date has amply shown that structured checklists that include as many objective details as possible (such as which specific history questions were asked and which were not) are much less prone to various implicit biases.

Finally, regardless of the findings, the authors are encouraged to present solutions to address racial bias, not only through training sensitivity training, but potentially by automating the assessment, possibly using artificial intelligence.

VERSION 1 - AUTHOR RESPONSE

Reviewer: 1

1. Choice of candidates. It's unclear why there are 3 white candidates but only 1 each of the non-white candidates. Could there not have been more non-white candidates? I also question the choice of Asian, Black and Chinese. Is Chinese not Asian? Of course Asian could mean many things, and is a very diverse group. Why not latino or middle eastern?

We thank the reviewer for their comment. We needed to have three white candidates for every non-white candidate, so that an SP assessor would see each non-white race paired with a different white candidate (the controls). We did not have the resources, nor could we have recruited sufficient SP assessors, to include more pairs of white/non-white candidates. Whilst we needed three white candidates and one for each non-white race to make the study design work, we have included the reliance on a single candidate of each non-white race as a limitation of the study (please see page 15, lines 7-9).

We selected two Asian subgroups, Chinese and South Asian. These two subgroups comprise the majority of Asian medical students in the UK, and we know that there are differences in the medical school awarding gap between these two groups. However, we appreciate that we should have been clearer as regards how we described the Asian group. We used 'Asian' as an accepted UK shorthand for South Asian (within the UK medical school student population, this group predominately comprises Indian, Pakistani and Bangladeshi students). We restricted our study to three non-white groups because again we had to make a compromise between the number of subgroups and our sample size of SP assessors/the time burden of participation. Nevertheless, it would clearly be beneficial to extend the study to include other racial groups and we have highlighted this in the revised manuscript.

2. It was also noted that the Asian and Black candidates did not have an accent when speaking but that the Chinese candidate did. This seems like a poor choice of candidates, and makes it difficult to state that race was the sole explanation for the difference, when attitudes about language fluency and intelligibility would also play a role here.

We thank the reviewer for raising this point. Although all candidates spoke English fluently, the Chinese candidate had a mild Chinese accent. We have included this as a limitation in the revised manuscript, and highlighted that the separate impacts of visual and aural racial cues could not be determined (please see page 15, lines 9-11).

4. Was there sufficient sample size to distinguish between the 3 non-white groups?

There was not sufficient sample size to distinguish between the three non-white groups and thus we do not make comparisons between the three non-white groups as this was not the aim of our study. No comparisons were possible due to our study design; SP assessors only saw one non-white candidate at a particular performance level. We apologise that our study intentions were not sufficiently clear, and we have now clarified this.

5. Regarding Table 1: I understand the rationale behind using median and IQR in this case. But I wonder if it glosses over small differences. Mean and SD might be more informative here. Also, a graphical display, e.g. a stacked barplot, might be a nice addition to understand the data.

We thank the reviewer for their helpful comment. We did not show mean and SD because the data were skewed, and therefore these summary statistics would be biased estimates of

location and spread. However, in the revised manuscript, we have added a box and whisker plot (Figure 2), which provides the detail that can be hidden by the use of median and IQR as the reviewer rightly points out. We have shown the mean difference in each box (please see page 11, Figure 2).

6. I found the category names hard to follow. Perhaps B1, B2, and B3 or something like that? or change "BD" for borderline to something else.

We apologise that our category names were confusing. We have spelled these out in the revised version of our paper.

7. "$p < 0.00057$" should probably contain an "="?
We used $p<0.0057$ as we are stating the p-value required for statistical significance after controlling for multiple comparisons.

8. DEI/EDI usually contains the word equity not ethnicity doesn't?
We apologise for the typo in the original manuscript. We have now corrected this (please see page 14, lines 15-17).

Reviewer 2

1. Title, typo “standardized”, “randomized”

We apologise for the typos. These have now been corrected.

2. Introduction is too redundant. It is unclear what is known and what is unknown, and what is the aim of this study. There is no clear statement why author performed this study.

We thank the reviewer for their comment, which has helped us improve the manuscript. We have revised the introduction to make it shorter. We have summarised the mixed evidence in the literature with respect to the effect of candidate race on scores awarded by SP assessors. We have also clarified our aims in the revised manuscript. We aimed to investigate the potential influence of candidate race on the scores awarded by SPs across various performance grades. (please see page 5, lines 15-19).

3. Asia and Chinese look similar (China is located in Asia), what is the reason authors compared A and C separately.

We apologise that this was not made clear in the original manuscript. We selected two Asian subgroups, Chinese and South Asian, as these two subgroups comprise the majority of Asian medical students in the UK, and we know that there are differences in the medical school awarding gap between these two groups. However, we appreciate that we should have been clearer as regards how we described the Asian group. We used 'Asian' as an accepted UK shorthand for South Asian (within the UK medical school student population, this group predominately comprises Indian, Pakistani and Bangladeshi students).

4. Statistical method should be discussed, this reviewer is unsure Wilcoxon matched pairs signed rank test was appropriate in this study.
   Also, there might be a problem in comparison, answer list in mark sheet should not be converted to discrete variable directly (0-8).

We apologise that this was not made clear in the original manuscript. We have now explained why we used the Wilcoxon matched pairs signed rank test (the data are discrete/skewed, and each white-non-white pair of candidates creates a pair). We agree there are controversies regarding the use of numerical values from qualitative descriptors. However, this is frequently done in practice and in research, and it is our view that providing the data are viewed as discrete rather than continuous, then numerical values can be applied without introducing bias.

5. There is no reason why authors compared white and non-white separately (fail, borderline, and pass).

We apologise we were not sufficiently clear. We did not compare white and non-white separately – indeed, comparing white to non-white was the aim of our study. We did the comparison of white to non-white at three levels of performance (fail, borderline and pass) as we hypothesised that the extent of racial bias could be influenced by performance level.

6. A various bias of simulated patients was not under consideration for comparison. For instance, when SPs were white, they might feel sympathy for white candidate. Furthermore, potential bias except for race such as behaviour, appearance, looks, etc should be discussed and omitted as much as possible.

Thank you for this excellent point. We agree that we were unable to analyse the impact of SP race; we simply did not have the resources to recruit sufficient SP assessors to enable us to do this. We have now discussed this as part of our future scope section (please see page 16,

lines 1-2). We controlled for candidate gender but were unable to control for all variables, and we have noted this as a limitation of our study (please see page 15, line 11-13).

7. Supplement is duplicated. In addition, the legend in supplement file should be attached.

We apologise for the duplication and lack of clarity. We have revised the supplemental material.

8. In discussion "The aim of this study was … communication and professional skills …the candidate". However, as authors stated, it is well known that race affect scores evaluated by assessors. Further information in detail about the aim of this study is necessary.

We thank the reviewer for their helpful comment. We have now clarified the aim in the discussion (please see page 12, lines 8-10). There is mixed evidence with respect to the effect of candidate race on scores awarded by SP assessors in examinations. Results may be impacted by context, including the grade at which the student is performing. We therefore aimed to investigate the potential influence of candidate race on the scores awarded by SPs to medical students performing at various grades.

9. Some of the sentences were repeated, should be avoided.

We apologise for this. We have made the revised manuscript more concise and avoided repetition.

10. "Our study found that the SPs gave significantly lower scores to the BF candidate as compared to the WF candidate" "the BBD and CBD candidates were given higher scores than the WBD candidate", there is no explanation why these results were obtained in this study. Theoretical explanation is needed in discussion. Authors are just guessing without any supported evidence/data.

We thank the reviewer for their comments. The sentences in the quote above are supported by the statistical analyses of the data in our study. We have provided potential reasons for these results in the discussion, but we cannot know for sure what the underlying reasons were. We were unable to find any existing literature regarding different biases at different performance levels/grades, hence the novelty of our work.

11. Overall, text and statistical method is complicated and involved sentence, more simple expression is preferable.

Thank you for highlighting; we have now revised the 'Statistical Methods' section.

Reviewer 3

1. Both the lack of similar findings for other races, as well as the very unusual finding of apparent reverse discrimination in the case of borderline students is difficult to explain. I am personally not convinced that the authors' explanation of this last finding is tenable — ie that racial bias is moderated by the standardised patients in the specific case of borderline students — nor can any solid scientific conclusions be unfortunately drawn from this.

   We thank the reviewer for their comments. We were also surprised by the study's findings and we can only hypothesise as to potential reasons. We have made it clearer in the revised discussion that we are suggesting possible explanations for our findings rather than having evidence for them. We agree further research is required to ascertain reasons for our findings.

2. Ultimately, the limited findings of the study may point back to study limitations which were at least in part noted by the authors themselves. Despite the large number of videos included — as required by the sample size calculation for the randomization — the presence of only one person from each race definitely opens the results to validity questions.

   We are grateful to the reviewer for highlighting this pertinent point. We agree that having one candidate of each race limits the generalisability of our findings. We have acknowledged this as a limitation of our study (please see page 15, lines 7-9).

3. It is also surprising that all students were male, and this unusual choice is not explained in the methodology.

   We apologise that this was not made clear in the original manuscript. It was important to mitigate against gender bias. Therefore, we needed to include candidates of the same gender. We have now added that further studies could assess the impact of both candidate race and gender (please see page 16, lines 2-3).

4. Another question which remains unanswered is the role of standardised patients' demographics on their ratings. While these characteristics are described in the supplementary information, and there is, as expected, a certain unbalanced distribution of races with the white one being predominant, no conclusions can be drawn on this important factor in the absence of any inferential or even descriptive analyses.

   We thank the reviewer for this excellent point. We agree that we were unable to analyse the impact of SP race or other demographics; we simply did not have the resources to recruit sufficient SP assessors to enable us to do this. We have now added this as part of our future scope section (please see page 16, lines 1-2).

5. The ultimate question regarding this study is its significance and impact. If the unusual finding regarding existing literature, unfortunately I do not see what they actually add to the literature.

   In the revised manuscript we have clarified the context and aim of the current study, and stipulated what our work is adding to the literature on awarding gaps. To date, there is mixed evidence with respect to the effect of candidate race on scores awarded by SP assessors in examinations. Some studies have demonstrated no effect on overall scores and interpersonal skills scores, whilst others have shown positive bias by SPs towards white students and lower overall scores for black/African American students. We explored whether patient scores are influenced by context, including the grade at which the student is performing. We therefore aimed to investigate the potential influence of candidate race on the scores awarded by SPs to medical students **performing at various grades**.
   Our results show that SPs seem to give non-white candidates performing at a borderline grade the benefit of the doubt, whereas non-white candidates performing at a fail grade receive lower scores than white students. These findings have important implications for SP assessor training and help raise awareness of potential biases when encountering candidates of different races performing at various grades.

6. I suspect that for the study to be practical, its size would need to be increased in terms of students actually being assessed, it would need to include more than one gender, correlate the findings with the demographics of the standardised patients themselves, and include a qualitative exploration of the reason why ratings were those observed.

   We agree that larger multi-centre studies are required to address the limitations of our study, and that all of the reviewers' suggestions are worthwhile avenues for further

research. We have acknowledged these points in the revised discussion (please see page 15, lines 7-9 and page 16, lines 1-3). Nevertheless, we believe that our findings, in this prospective randomised study, can help raise awareness of potential biases that may exist in relation to candidates of different races performing at various grades.

7. Another area that requires exploration in light of this study is the type of assessments used by standardised patients. In the current study all of these assessments are purely subjective, almost "inviting" almost any inherent implicit biases to come out. Educational literature to date has amply shown that structured checklists that include as many objective details as possible (such as which specific history questions were asked and which were not) are much less prone to various implicit biases.

   We thank the reviewer for their comments. We aimed to focus on communication, and professional skills, and global impression. Therefor the focus was on the SP's impression of the soft skills within a consultation as opposed to the medical content. We used 'real life' mark sheets, similar to those used in other UK medical schools. How SPs should rate candidates is another valid research question. While we agree structure is important, patients often report 'gut feel' for a clinician, and their 'perception of the communication' within the consultation predicts satisfaction (Ha, J. F., and Longnecker, N. (2010). Doctor-Patient Communication: A Review. Ochsner J. 10, 38-43.). These principles underpinned the mark sheet design used in our study.

8. Finally, regardless of the findings, the authors are encouraged to present solutions to address racial bias, not only through training sensitivity training, but potentially by automating the assessment, possibly using artificial intelligence.

We thank the reviewer for their suggestion. We have considered other possible avenues for addressing racial bias. Whilst at present AI is unable to mark candidates' communication and professional skills, this is an area that may evolve in future.

VERSION 2 - REVIEW

Reviewer 1

Name Haile, Sarah

Affiliation University of Zurich, Epidemiology, Biostatistics and Prevention Institute

Date **24-Oct-2024**

COI

Thank you for this revision, which has addressed most of my concerns.

I am still stuck a bit on the use of the term "Asian" here, and would argue that it has the tendency to be misunderstood here. While the authors come from the UK, this is not a major point of the paper, and Asian would not necessarily be understood to be coming from a UK context. I come from the US, where Asian can mean anything from say South Asian to Chinese to Vietnamese or Indonesian. It also appears that recent UK census questionnaires use Asian to mean a range of backgrounds including Chinese (https://www.ethnicity-facts-figures.service.gov.uk/style-guide/ethnic-groups/).

With that in mind, I would suggest the following minor edits: 1) in the abstract, write the categories as "South Asian, black and Chinese". 2) In the methods section under Variables and Data Measurement where it states "three candidates were from the non-white group consisting of Asian, black and Chinese ethnicities" (bottom of page 6), change it to "three candidates were from the non-white group consisting of Asian (also referred to as South Asian, indicating people of Indian, Pakistani or Bangladeshi descent), black and Chinese ethnicities" or similar.

VERSION 2 - AUTHOR RESPONSE

Reviewer: 1
Dr. Sarah Haile, University of Zurich
Comments to the Author:
Thank you for this revision, which has addressed most of my concerns.

I am still stuck a bit on the use of the term "Asian" here, and would argue that it has the tendency to be misunderstood here. While the authors come from the UK, this is not a major point of the paper, and Asian would not necessarily be understood to be coming from a UK context. I come from the US, where Asian can mean anything from say South Asian to Chinese to Vietnamese or Indonesian. It also appears that recent UK census questionnaires use Asian to mean a range of backgrounds including Chinese (https://www.ethnicity-facts-figures.service.gov.uk/style-guide/ethnic-groups/).

With that in mind, I would suggest the following minor edits:

1) in the abstract, write the categories as "South Asian, black and Chinese".

Response: We thank the reviewer for their very valid point. We have amended the abstract as advised. (please see page 2, line 11 of the revised manuscript).

2) In the methods section under Variables and Data Measurement where it states "three candidates were from the non-white group consisting of Asian, black and Chinese ethnicities" (bottom of page 6), change it to "three candidates were from the non-white group

consisting of Asian (also referred to as South Asian, indicating people of Indian, Pakistani or Bangladeshi descent), black and Chinese ethnicities" or similar.

Response: We are grateful to the reviewer for their excellent suggestion. We have included their helpful clarification in the revised manuscript (please see page 6, lines 20-22 of the revised manuscript).

VERSION 3 - REVIEW

Reviewer 1

Name Haile, Sarah

Affiliation University of Zurich, Epidemiology, Biostatistics and Prevention Institute

Date 25-Nov-2024

COI

No further comments.
